# Correct use and ease of use of a placebo dry powder inhaler in subjects with asthma and chronic obstructive pulmonary disease

**DOI:** 10.1177/1479973118815692

**Published:** 2018-12-18

**Authors:** Gregory J Feldman, Dmitry V Galkin, Pinal Patel, Kathryn A Collison, Raj Sharma

**Affiliations:** 1 S. Carolina Pharmaceutical Research, Spartanburg, SC, USA; 2 Respiratory Medical Franchise, GlaxoSmithKline, Durham, NC, USA; 3 Respiratory Therapy Area Unit, GlaxoSmithKline, Uxbridge, UK; 4 Respiratory Medical Franchise, GlaxoSmithKline, Brentford, UK

**Keywords:** Asthma, chronic obstructive pulmonary disease, dry powder inhaler, ELLIPTA, ease of use, inhaler errors

## Abstract

Correct use and ease of use of a placebo dry powder inhaler was evaluated in two single-arm, United States-multicenter, phase-IV studies in adults with asthma (*n* = 259) or chronic obstructive pulmonary disease (COPD; *n* = 278) who were receiving maintenance inhaler therapy. Subjects demonstrating correct placebo inhaler use within three attempts at screening were instructed to take once-daily inhalations from the inhaler for 28 ± 2 days (continuing usual maintenance), followed by randomization to complete one of two versions of an ease-of-use questionnaire and reassessment for correct inhaler use. At study end, 96% asthma/93% COPD subjects rated the placebo inhaler as “easy” or “very easy” to use while demonstrating correct use. Furthermore, 99% asthma/99% COPD subjects indicated it was “easy” or “very easy” to determine number of doses remaining, and 81%/84%, respectively, indicated they would be “likely” or “very likely” to request their current medication in the inhaler, if available. Adverse event (AE) rates were 12% asthma/15% COPD, most frequently headache (3%/3%). Treatment-related AEs were reported in one subject with asthma (cough) and four subjects with COPD (cough, *n* = 3; back pain, *n* = 1). At study end, most subjects with asthma or COPD operated the placebo inhaler correctly and found it easy to use.

## Introduction

The mainstay of treatment for obstructive respiratory diseases involves inhaled medications,^1^ including inhaled corticosteroids for asthma^2^ and bronchodilators for chronic obstructive pulmonary disease (COPD).^3^ Inhaled medications for asthma and COPD are available in a variety of delivery systems, including pressurized metered-dose inhalers (MDIs) and dry powder inhalers.^4^ Each inhaler has unique features and instructions for use, and as such, inhalers vary in the technique required for proper use and no standard survey exists to evaluate correct use. For effective self-delivery of inhaled medications, inhalers should be easy to operate correctly by subjects.

The choice of inhalation device is an important consideration because it can potentially affect long-term outcomes.^3^ Incorrect inhaler technique can be associated with decreased efficiency in delivery of the medication (which in turn may lead to poor efficacy), poor disease control, and reduced treatment compliance.^5–10^ Data from previous studies suggest that a substantial proportion of subjects with asthma or COPD use their inhalers incorrectly.^5,6,9,10^

The multidose dry powder inhaler, ELLIPTA (GSK, Brentford, Middlesex, UK), was developed for the delivery of a range of once-daily inhaled medications for asthma and COPD. This inhaler can accommodate one or two blister strips, with each blister containing a sealed single dose of medication. The single-strip and double-strip configurations allow for aerosol delivery of monotherapies and combination therapies, respectively, with medications stored separately until the point of administration.^11^ This dry powder inhaler is currently approved for use with fluticasone furoate (Arnuity), fluticasone furoate/vilanterol (Relvar/Breo), umeclidinium bromide/vilanterol (Anoro), and umeclidinium bromide (Incruse).

In a previous pooled analysis of data from three multicenter, randomized trials that assessed fluticasone furoate/vilanterol combination therapy and/or fluticasone furoate monotherapy in subjects with asthma, in which the dry powder inhaler was used to deliver study medication, 94% of subjects rated the inhaler as “easy” or “very easy” to use.^12^ In a separate study that assessed asthma and COPD subjects’ experience of the dry powder inhaler using a semi-structured, in-depth, qualitative interview approach, the inhaler was frequently described by participants as straightforward to operate and easy to use.^13^ Furthermore, in a recent open-label, randomized study that assessed inhaler errors and inhaler preference in subjects with asthma or COPD, significantly higher proportions of subjects rated the dry powder inhaler as “very easy” or “easy” to use compared with other inhalers.^9^

We conducted two single-arm, phase-IV clinical studies to evaluate the correct use and ease of use of a placebo version of the dry powder inhaler in adult subjects with asthma or COPD. Importantly, these studies were specifically designed to assess ease of use of the placebo inhaler in subjects who were determined to operate the inhaler correctly.

## Methods

### Subjects

In the asthma study, eligible subjects were males or females aged ≥18 years who had an established diagnosis of asthma according to the National Institutes of Health 2007 criteria^14^ and who were receiving maintenance therapy for asthma, but with no prior or ongoing use of the dry powder inhaler. In the COPD study, eligible subjects were males or females aged ≥40 years who had an established diagnosis of COPD according to the American Thoracic Society/European Respiratory Society 2004 guidelines^15^ and who were receiving maintenance inhaler therapy for COPD, but with no use of the dry powder inhaler within the previous 6 months. Full inclusion/exclusion criteria for the asthma and COPD studies are provided in the Online Appendix A.

The protocols were approved by institutional review boards/independent ethics committees for the individual participating centers, and the trials were conducted in accordance with the ethical principles founded in the Declaration of Helsinki and Good Clinical Practice Guidelines. All subjects provided written informed consent.

### Study design and assessments

Both studies were 28-day, single-arm, open-label, randomized, US-multicenter, phase-IV trials conducted to assess the correct use and ease of use of a placebo version of the dry powder inhaler in subjects with asthma (GSK study 201594/NCT02586506) or COPD (GSK study 201071/NCT02586493).

Each trial comprised two study visits and a telephone call. At visit 1 (screening; day 1), eligible subjects with asthma or COPD were required to demonstrate correct use of the placebo dry powder inhaler within three attempts. Prior to their first attempt, subjects reviewed written instructions for the correct use of the inhaler based on the patient information leaflet (see the Online Appendix B), but did not receive any training. Subjects were permitted to receive training (verbal instruction and a demonstration of correct use) from the inhaler-trained health-care professional (HCP) in between attempts 1 and 2, and/or attempts 2 and 3, if necessary. Correct use was evaluated by the HCP using a Correct Use Checklist (see the Online Supplementary Table 1). Subjects unable to demonstrate the correct use of the placebo inhaler within three attempts at visit 1 were considered screening failures and did not continue in the study.

Subjects who demonstrated the correct use of the placebo inhaler at visit 1 were instructed to take once-daily inhalations from the inhaler for 28 ± 2 days, while continuing with their usual asthma or COPD maintenance therapy. Safety was assessed by the HCP through adverse event (AE) monitoring and subjects’ completion of a Medical Problems/Medications Taken Worksheet. Subjects received the Medical Problems/Medications Taken Worksheet at visit 1 and were instructed to record any medical problems experienced or any change in existing medication(s) throughout the course of the study. Safety assessments were conducted at visit 1, on day 8 ± 2 (by telephone), and at visit 2 (study end) or early withdrawal.

At visit 2 (study end; day 28 ± 2), subjects in each study were randomized 1:1 by investigators using a Registration and Medication Ordering System to complete one of two versions of an ease-of-use questionnaire (see the Online Appendix C). Easy to use was defined as a rating of “easy” or “very easy” on a four-point Likert scale (very easy, easy, difficult, or very difficult). The two versions of the questionnaire differed only in the order of listing of the four rating categories. At study end, subjects were also reassessed by the HCP for the correct use of the placebo inhaler in a single attempt without any additional instruction.

### Endpoints

The primary endpoint in both studies was the percentage of subjects who rated the use of the placebo dry powder inhaler as “easy” or “very easy,” among those who demonstrated the correct use of the inhaler at study end. Secondary endpoints were the percentage of subjects who demonstrated the correct use of the placebo inhaler at study end and the percentage of subjects who rated the ability to determine the number of doses remaining in the placebo inhaler as “easy” or “very easy” at study end. Exploratory endpoints included the percentage of subjects who indicated that they would be “likely” or “very likely” to ask their doctor for the dry powder inhaler if their current daily inhaled asthma/COPD medication(s) were available in this inhaler, the percentage of subjects who demonstrated the correct use of the placebo inhaler after reading the directions only (visit 1, attempt 1), and the percentage of subjects who demonstrated the correct use of the placebo inhaler after reading the directions and receiving training (visit 1, attempts 2 and 3, assessed separately).

### Statistical analyses

There were no formal calculations of power or sample size for these studies. In each study, it was planned to randomize 239 subjects with an expectation that 208 subjects (104 subjects each randomized to complete ease-of-use questionnaire version A or B) would complete the study and demonstrate correct use of the placebo inhaler at study end. Data from the two studies were collected and analyzed separately. In both studies, demographics/baseline characteristics, analysis of correct use of the placebo inhaler at screening, and safety were assessed in the intention-to-treat (ITT) population, which comprised all subjects who were screened and who received at least one dose of placebo study treatment. Analyses of prespecified endpoints on correct use and ease of use were performed in the modified ITT (mITT) population; this included all subjects, who were screened, received at least one dose of placebo study treatment, and were randomized to receive the ease-of-use questionnaire at study end.

## Results

### Study conduct

The asthma study was conducted at 15 investigational sites in the United States between October 22, 2015 and February 4, 2016, and the COPD study at 17 sites in the United States between October 22, 2015 and March 15, 2016.

### Subjects

**Figure 1.**
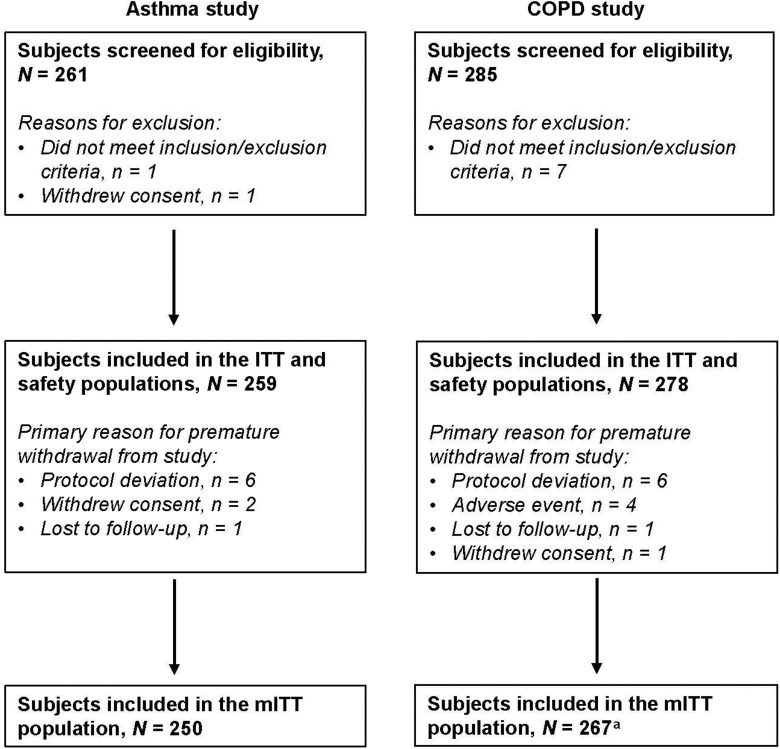
Subject flow through the asthma and COPD studies. ^a^One subject in the COPD study was withdrawn early and randomized in error; the ease-of-use questionnaire was not administered. COPD: chronic obstructive pulmonary disease; ITT: intention-to-treat; mITT: modified intention-to-treat.

In total, 261 asthma subjects and 285 COPD subjects were screened for eligibility; of these, 259 and 278 subjects, respectively, who met the inclusion criteria and received at least one dose of placebo study treatment were included in the respective ITT populations. A summary of subject flow through the studies is provided in Figure 1.

Subject demographics and baseline characteristics are summarized in Table 1. In the asthma study, subjects’ mean age was 47.3 years and 56% had an extensive asthma history of ≥20 years. In the COPD study, subjects’ mean age was 64.8 years and 35% had a COPD history of ≥10 years.

**Table 1.** Subject demographics and baseline characteristics (intention-to-treat populations).

Characteristic	Asthma study (*n* = 259)	COPD study (*n* = 278)
Age (years), mean (SD)	47.3 (15.23)	64.8 (8.56)
Male/female, *n* (%)	94 (36)/165 (64)	147 (53)/131 (47)
Race, *n* (%)^a^		
White	184 (72)	249 (90)
African American/African	64 (25)	28 (10)
American Indian/Alaska Native	4 (2)	1 (<1)
Asian	4 (2)	0
Native Hawaiian/other Pacific Islander	1 (<1)	0
BMI (kg/m^2^), mean (SD)^b^	31.3 (8.1)	28.7 (6.8)
Duration of asthma/COPD, *n* (%)^c^		
<1 year	5 (2)	0
≥1 to <5 years	24 (9)	87 (31)
≥5 to <10 years	26 (10)	95 (34)
≥10 to <15 years	20 (8)	52 (19)
≥15 to <20 years	39 (15)	32 (12)
≥20 to <25 years	33 (13)	4 (1)
≥25 years	112 (43)	8 (3)
COPD type, *n* (%)		
Chronic bronchitis^d^	NA	186 (67)
Emphysema^d^	NA	115 (41)
Missing	NA	9 (3)
Smoking history, *n* (%)^b^		
Never smoked	207 (80)	1 (<1)^e^
Former smoker	52 (20)	152 (55)
Current smoker	0	124 (45)
Smoking pack years, mean (SD)^f^	3.4 (3.1)	52.0 (27.2)

COPD: chronic obstructive pulmonary disease; BMI: body mass index; SD: standard deviation; NA: not applicable.

^a^ Data available for 257 subjects in the asthma study.

^b^ Data available for 277 subjects in the COPD study.

^c^ As relevant: duration of asthma for subjects in the asthma study and duration of COPD for subjects in the COPD study.

^d^ Subjects could select either category or both for COPD type.

^e^ Subject noted as a protocol violation.

^f^ Data available for 52 subjects (former smokers) in the asthma study and 276 subjects (former/current smokers) in the COPD study.

### Treatment compliance

Subject compliance with study inhaler use was assessed at the beginning and end of each study based on the dose counter on the placebo dry powder inhaler (ITT populations). In the asthma study, the mean overall compliance was 98.57% (standard deviation (SD) = 10.63), with the majority of subjects (*n* = 203, 78%) in the 95–105% compliance category. In the COPD study, the mean overall compliance was 97.82% (SD = 8.31), with the majority of subjects (*n* = 221, 79%) in the 95–105% compliance category. Few subjects were <80% compliant (*n* = 9, 3% of asthma subjects; *n* = 6, 2% of COPD subjects).

### Correct use and ease of use of placebo dry powder inhaler

**Figure 2.**
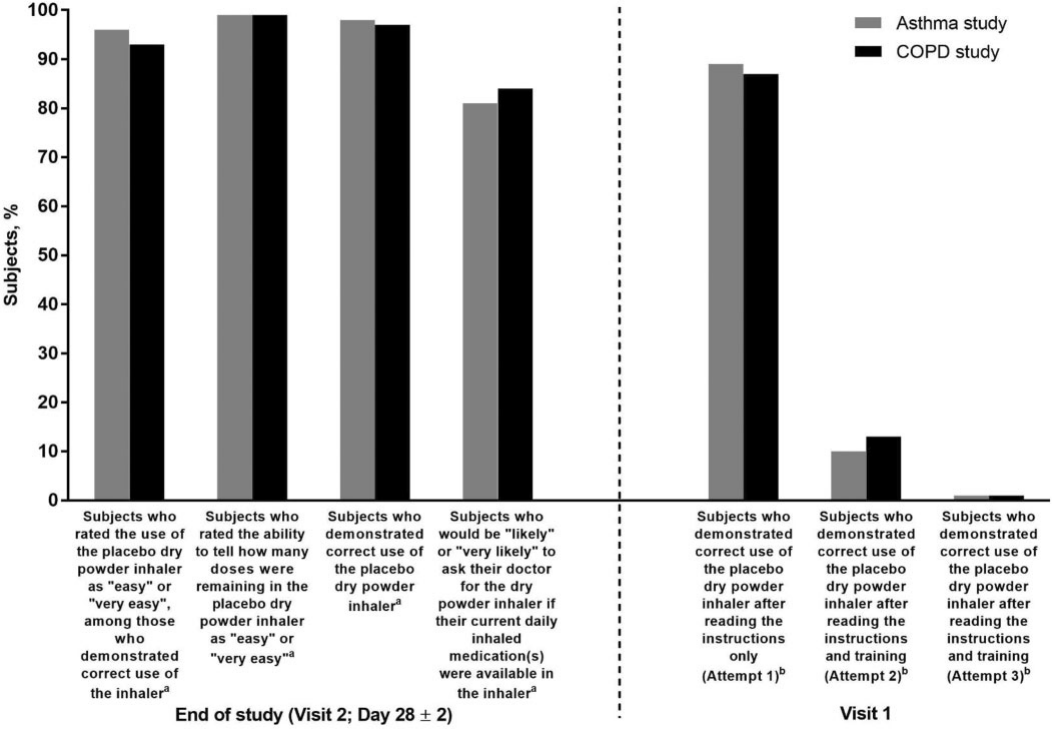
Key findings for correct use and ease of use of the placebo dry powder inhaler. ^a^Modified intention-to-treat population; ^b^intention-to-treat population.

Key findings on correct use and ease of use of the dry powder inhaler are summarized in Figure 2, and errors in subject technique are summarized in Tables 2 and 3.

**Table 2.** Errors in technique with the placebo dry powder inhaler in the asthma study.

Subjects with error in technique, *n* (%)^a^	Visit 1 (screening) (*n* = 259)		Visit 2 (study end) (*n* = 250)
	Attempt 1 (*n* = 28)^b^	Attempt 2 (*n* = 2)	Attempt 3 (*n* = 6)
Subject did not breathe out (exhale) while holding the inhaler away from his/her mouth	15 (54)	1 (50)	6 (100)
Subject blocked air vent with fingers	10 (36)	1 (50)	1 (17)
Subject did not close the inhaler completely	9 (32)	2 (100)	1 (17)
Subject did not remove the inhaler from his/her mouth and held his/her breath	9 (32)	2 (100)	1 (17)
Subject did not breathe out slowly and gently	7 (25)	1 (50)	1 (17)
Subject did breathe into the mouthpiece	5 (18)	1 (50)	4 (67)
Subject did not take one long steady deep breath in through his/her mouth	4 (14)	1 (50)	1 (17)
Subject did not place mouthpiece between lips and close lips firmly around it	3 (11)	1 (50)	1 (17)
Subject did shake the inhaler	3 (11)	0	1 (17)
Subject did not slide the cover completely down to expose the mouthpiece until a “click” was heard	2 (7)	0	1 (17)

^a^ Subjects could be counted in more than one error category per visit/attempt.

^b^ One subject demonstrated incorrect use at attempt 1 and chose not to continue with the remaining attempts.

**Table 3.** Errors in technique with the placebo dry powder inhaler in the COPD study.

Subjects with error in technique, *n* (%)^a^	Visit 1 (screening) (*n* = 278)		Visit 2 (study end) (*n* = 267)
	Attempt 1 (*n* = 36)	Attempt 2 (*n* = 1)	Attempt 3 (*n* = 8)
Subject did not breathe out (exhale) while holding the inhaler away from his/her mouth	17 (47)	0	7 (88)
Subject did breathe into the mouthpiece	8 (22)	0	3 (38)
Subject did not slide the cover completely down to expose the mouthpiece until a “click” was heard	7 (19)	0	1 (13)
Subject did not take one long steady deep breath in through his/her mouth	7 (19)	1 (100)	0
Subject did not remove the inhaler from his/her mouth and held his/her breath	6 (17)	1 (100)	1 (13)
Subject did not breathe out slowly and gently	5 (14)	1 (100)	0
Subject did not close the inhaler completely	3 (8)	0	0
Subject blocked air vent with fingers	3 (8)	0	0
Subject did shake the inhaler	2 (6)	0	1 (13)
Subject did not place mouthpiece between lips and close lips firmly around it	1 (3)	0	0

COPD: chronic obstructive pulmonary disease.

^a^ Subjects could be counted in more than one error category per visit/attempt.

In the asthma study, 231 (89%) subjects demonstrated the correct use of the placebo inhaler on the first attempt at the screening visit. Twenty-eight (11%) subjects made errors in technique on attempt 1, the most common error being failing to exhale while holding the inhaler away from the mouth (*n* = 15; Table 2). Two (<1%) subjects failed to demonstrate correct use at attempt 2 and no subjects demonstrated incorrect use at attempt 3.

In the COPD study, 242 (87%) subjects demonstrated the correct use of the placebo inhaler on the first attempt at the screening visit. Thirty-six (13%) subjects made errors in technique on attempt 1, most commonly failing to exhale while holding the inhaler away from the mouth (*n* = 17; Table 3). Only one (<1%) subject used the inhaler incorrectly on attempt 2 and no subjects demonstrated incorrect use on attempt 3.

A total of 250 subjects completed the asthma study and were randomized to receive the ease-of-use questionnaire; this comprised the mITT population for analyses of correct use and ease of use of the dry powder inhaler at study end. In the COPD study, 266 subjects completed the study; however, one subject who was prematurely withdrawn from the study was randomized in error but did not receive the ease-of-use questionnaire. Thus, the mITT population in the COPD study comprised 267 subjects. At the end of the 28-day study period, 244 (98%) asthma subjects and 258 (97%) COPD subjects demonstrated the correct use of the placebo inhaler within a single attempt (Figure 2). The most common observed errors in the six asthma subjects and eight COPD subjects who failed to demonstrate the correct use of the placebo inhaler at study end were failing to exhale while holding the inhaler away from the mouth (*n* = 6 and *n* = 7, respectively) and breathing into the mouthpiece (*n* = 4 and *n* = 3 subjects, respectively; Tables 2 and 3).

Among subjects demonstrating the correct use of the placebo inhaler at study end, 96% of asthma subjects and 93% of COPD subjects rated the inhaler as “easy” or “very easy” to use (Figure 2). In both studies, 99% of subjects indicated that it was “easy” or “very easy” to determine the number of doses remaining in the placebo inhaler. Furthermore, 81% of asthma subjects and 84% of COPD subjects indicated that they would be “likely” or “very likely” to request their current prescribed asthma/COPD medication(s) in the dry powder inhaler, if it were available. Findings were consistent between versions A and B of the ease-of-use questionnaire (data not shown).

### Safety

As these studies assessed correct use and ease of use of a placebo dry powder inhaler, no active drug was administered to subjects; however, subjects did continue with their usual asthma or COPD maintenance therapy throughout. In the asthma study, the most common on-treatment respiratory asthma medications were salbutamol/albuterol (77%), salmeterol xinafoate/fluticasone propionate (33%), and budesonide/formoterol fumarate (28%). The most common on-treatment respiratory COPD medications in the COPD study were salbutamol/albuterol (59%), tiotropium bromide (50%), and budesonide/formoterol fumarate (37%).

On-treatment AEs were reported in 30 (12%) asthma subjects and 43 (15%) COPD subjects; the most common AEs in the asthma study were headache (3%) and upper respiratory tract infection (2%), and in the COPD study were headache (3%) and nasopharyngitis (2%; Table 4). There were no reported events of on-treatment pneumonia in either study. Treatment-related AEs were reported in one asthma subject (cough) and four COPD subjects (cough, *n* = 3; back pain, *n* = 1). No serious AEs, fatal AEs, or AEs leading to withdrawal or permanent discontinuation of study treatment were reported in the asthma study. Serious AEs of COPD exacerbation were reported in four subjects in the COPD study; none of these events was fatal or treatment-related, but all led to subject withdrawal.

**Table 4.** On-treatment adverse events occurring in >1 subject in either study (intention-to-treat populations).

Subjects, *n* (%)	Asthma study (*n* = 259)	COPD study (*n* = 278)
Any adverse event	30 (12)	43 (15)
Headache	9 (3)	8 (3)
Upper respiratory tract infection	5 (2)	1 (<1)
Cough	3 (1)	3 (1)
Nasopharyngitis	3 (1)	6 (2)
Oropharyngeal pain	3 (1)	0
Urinary tract infection	2 (<1)	0
Arthralgia	1 (<1)	3 (1)
Back pain	1 (<1)	4 (1)
Sinusitis	1 (<1)	4 (1)
Exacerbation of COPD	0	4 (1)
Rash	0	2 (<1)

COPD: chronic obstructive pulmonary disease.

## Discussion

Features of an inhaler considered most important to subjects include the overall ease of use, dose counter, and easy to learn to use.^16,17^ Although prior studies have assessed subjects’ perception of the ease of use of this dry powder inhaler,^9,12,13,18^ ease of use ratings in subjects determined to be correctly operating the inhaler has not previously been adequately addressed. The present phase-IV trials were conducted to assess the ease of use of a placebo version of the dry powder inhaler only in subjects who were determined to be using the inhaler correctly.

Previous studies in subjects with asthma and COPD have demonstrated low critical and overall error rates with this dry powder inhaler.^9,11,18,19^ In contrast, higher error rates have been reported with other inhalation devices, including MDIs, Diskus, Turbuhaler, and Handihaler.^7,9,17,20–25^ Consistent with previous reports,^9,11,12,18^ high proportions of asthma and COPD subjects in the present studies were able to demonstrate the correct use of the placebo inhaler on their first attempt at the screening visit, after reading the instructions for use only. At the end of the 28-day study period, almost all asthma and COPD subjects were still able to demonstrate the correct use of the inhaler in a single attempt without any additional training, a finding in line with other data in the literature supporting favorable retained correct use of this dry powder inhaler.^12,13,18,19^

Among subjects determined to be correctly operating the placebo inhaler at study end, the majority found the inhaler easy to use. Almost all subjects found it easy to determine the number of doses remaining in the inhaler and a high proportion of subjects in both studies indicated that they would be likely to request their regular prescribed asthma/COPD medication(s) in the dry powder inhaler from their doctor, if it were available.

In both the asthma and COPD studies, the most common error in technique with the placebo inhaler at screening and at study end was a failure to exhale while holding the inhaler away from the mouth; notably, this was also the most common error with the use of this dry powder inhaler in a recent randomized cross-over study of inhaler errors and inhaler preference in subjects with asthma and COPD.^9^

AE rates in both the asthma and COPD studies were low, and treatment-related AEs were very infrequent; this is perhaps not surprising considering that these studies evaluated a placebo version of the dry powder inhaler and no active drug was administered to subjects. The observed AEs were most likely related to subjects’ ongoing prescribed therapy for asthma or COPD. The most common on-treatment respiratory medications for subjects in the present studies were salbutamol, salmeterol xinafoate/fluticasone propionate, budesonide/formoterol fumarate, and tiotropium bromide, and the most common reported AEs (headache, upper respiratory tract infection, nasopharyngitis) are among the known side effects of these medications.

It is possible that certain AEs associated with the delivery of medications via the dry powder inhaler would not have been captured, or their incidence underestimated, in our studies. For example, dry mouth and sore throat are among the common side effects associated with the use of Relvar/Breo ELLIPTA in subjects with asthma and COPD.^26,27^ One analysis of the frequency of side effects associated with Breo ELLIPTA, based on reports to the U.S. Food and Drug Administration, found that 1.4% of people reporting side effects (85/6059) experienced a dry mouth,^28^ while “pain and irritation in the back of the mouth and throat” has been estimated to affect up to 1 in 10 subjects taking Relvar ELLIPTA.^27^ These side effects are likely related to the deposition of medication during inhalation, which may explain why they were not commonly reported in our study.

Treatment compliance was high in our study (mean compliance rates of 98.57% and 97.82% for asthma and COPD subjects, respectively), which is in line with a recent report demonstrating higher adherence rates with the placebo ELLIPTA dry powder inhaler versus two different comparator placebo MDIs in subjects with asthma (mean adherence rates of 95.2% and 93.4% for ELLIPTA vs. MDI-1 and 87.9% and 85.9% for ELLIPTA vs. MDI-2).^29^ These together with our other findings suggest that the ease with which subjects are able to correctly operate ELLIPTA dry powder inhaler may be a contributing factor in facilitating treatment compliance/adherence.

A key advantage to the design of these studies was that subjects were able to continue with their existing prescribed asthma/COPD treatment while the placebo inhaler was evaluated over time without the confounding factors of another active medication. While our results indicate that the majority of asthma and COPD subjects in the present studies found the placebo inhaler easy to use, there are limitations of this analysis that should be considered in interpretation of the findings. Both studies were multicenter trials conducted solely in the United States and caution should be taken in extrapolating findings to other countries. Furthermore, the open-label design of the studies involving trained HCPs’ subjective assessments of the correct use of the placebo inhaler introduces the potential for bias. Subjects included in these studies received more thorough instruction/training on the use of the inhaler than they would have likely received in the routine clinical practice setting; thus, it is possible that subjects’ perception of the ease of use of the placebo inhaler may have been overestimated. In the COPD study, due to initial difficulties in identifying subjects who had no prior experience with the dry powder inhaler, a protocol amendment was implemented to permit recruitment of subjects who had not used the dry powder inhaler within 6 months prior to screening; we cannot therefore discount the possibility that ease-of-use findings in this study may have been impacted by COPD subjects’ previous experience with the dry powder inhaler outside of this 6-month window. Finally, while a strength and novel aspect of the study design was assessing ease of use of the placebo dry powder inhaler in subjects deemed to be correctly operating the inhaler, it could be argued that a potential subject selection bias may have been introduced as a result. However, it is of note that all 14 subjects (*n* = 6 asthma and *n* = 8 COPD) who demonstrated the incorrect use of the placebo inhaler in the single attempt at study end rated the inhaler as “easy” or “very easy” to use (data not shown).

## Conclusions

Overall, these results demonstrate that the majority of asthma and COPD subjects without previous experience of the dry powder inhaler were able to operate the inhaler correctly and found it easy to use.

## Supplemental material

Supplemental Material, CRD-18-0043_Feldman_et_al_supplement_09Oct18 - Correct use and ease of use of a placebo dry powder inhaler in subjects with asthma and chronic obstructive pulmonary disease Click here for additional data file. Supplemental Material, CRD-18-0043_Feldman_et_al_supplement_09Oct18 for Correct use and ease of use of a placebo dry powder inhaler in subjects with asthma and chronic obstructive pulmonary disease by Gregory J Feldman, Dmitry V Galkin, Pinal Patel, Kathryn A Collison, and Raj Sharma in Chronic Respiratory Disease
