# Supplementary material for: Correct use and ease of use of a placebo dry powder inhaler in subjects with asthma and chronic obstructive pulmonary disease
Source: Chron Respir Dis. 2018 Dec 18;16:1479973118815692. doi: 10.1177/1479973118815692 (PMC6302980; doi:10.1177/1479973118815692)
Supplement: Supplemental Material, CRD-18-0043_Feldman_et_al_supplement_09Oct18 - Correct use and ease of use of a placebo dry powder inhaler in subjects with asthma and chronic obstructive pulmonary disease [file CRD-18-0043_Feldman_et_al_supplement_09Oct18.pdf]

Online supplementary material

Appendix A. Key inclusion/exclusion criteria for the asthma and COPD studies.

	Asthma study	COPD study
**Key inclusion criteria**	Aged ≥18 y	Aged ≥40 y
	Established diagnosis of asthma (NIH 2007 criteria)[a]	Established diagnosis of COPD (ATS/ERS 2004 guidelines)[b]
	Demonstrated historical reversibility of ≥12% and ≥200 mL reversibility of FEV_1 within 10–40 min following 2–4 inhalations of salbutamol/albuterol inhalation aerosol (or equivalent nebulized treatment with salbutamol/albuterol solution) within 24 months of Visit 1	Post-bronchodilator FEV_1 of ≤70% of predicted and FEV_1/FVC ratio of <0.70
	Receiving maintenance inhaler therapy for asthma	Receiving maintenance therapy for COPD
	No prior or ongoing use of the ELLIPTA dry powder inhaler	No use of the ELLIPTA dry powder inhaler within previous 6 months
	Capable of continuing with current prescribed asthma maintenance inhaler therapy and SABA for rescue use as needed throughout the study	Capable of continuing with current prescribed COPD maintenance therapy and SABA for rescue use as needed throughout the study
		Current or former smoker with a >10-pack-year smoking history
**Key exclusion criteria**	Current diagnosis of COPD	Current diagnosis of asthma

Any change/planned change of asthma treatment within 4 weeks prior to screening/Visit 1	Any change of COPD treatment within 4 weeks of screening/Visit 1
Any asthma exacerbation within 4 weeks, or any hospitalization or emergency department visit due to asthma within 3 months of screening/Visit 1	Any COPD exacerbation requiring systemic corticosteroids and/or antibiotics within 4 weeks, or hospitalization for COPD within 3 months of screening/Visit 1
Other respiratory disorders	Other respiratory disorders or other clinically significant disease abnormalities
Use of an investigational drug and/or medical device/inhaler within 30 days of screening/Visit 1, or within five drug half-lives of the investigational drug, whichever was longer	Lung volume reduction surgery within 12 months of screening/Visit 1
History of life-threatening asthma, defined as an asthma episode that required intubation and/or was associated with hypercapnia, respiratory arrest, or hypoxic seizures	Long-term use of oxygen therapy
Daily asthma treatment with inhaled SABA only (as needed or regularly scheduled)	
Poorly controlled or unstable asthma, which, in the investigator’s judgment, would affect the subject’s ability to	

	evaluate ease of use and correct use

ATS: American Thoracic Society; ERS: European Respiratory Society; NIH: National Institutes of Health; SABA: short-acting β_2 agonist.

[a]Asthma diagnosed according to the 2007 guidelines of the NIH[1]; [b]COPD diagnosed according to 2004 guidelines of ATS/ERS.[2]

Appendix B. Instructions for use.

**Wait to open the cover until you are ready to take your dose.**	
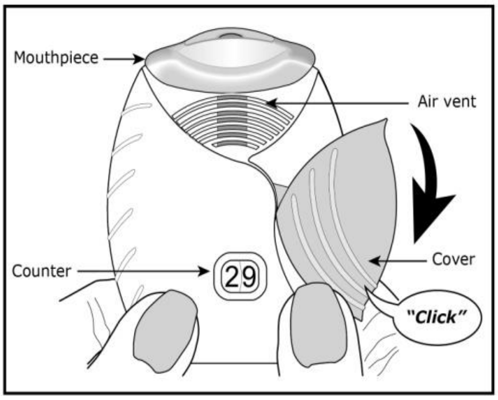<br>**Figure A**	**Step 1. Open the cover of the inhaler. See Figure A.**<br>• Slide the cover down to expose the mouthpiece. You should hear a "click." The counter will count down by 1 number. You do not need to shake this kind of inhaler. **Your inhaler is now ready to use.**<br>• If the counter does not count down as you hear the click, the inhaler will not deliver the dose. Call your healthcare provider or pharmacist if this happens.
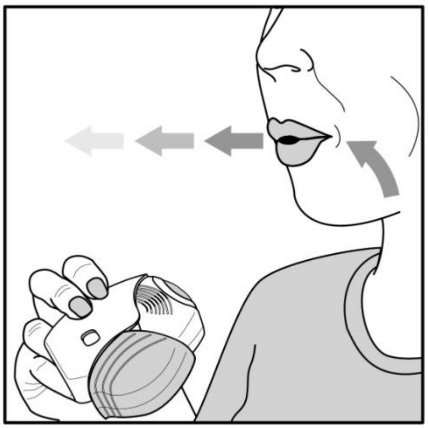<br>**Figure B**	**Step 2. Breathe out. See Figure B.**<br>• While holding the inhaler away from your mouth, breathe out (exhale) fully. Do not breathe out into the mouthpiece.

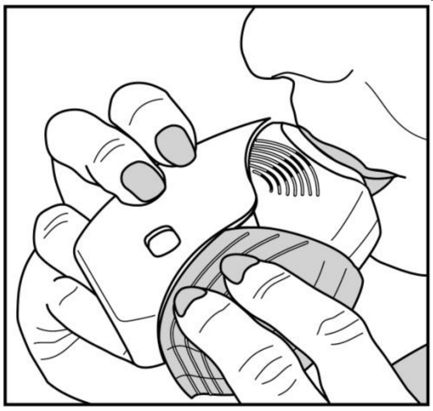<br>**Figure C**	**Step 3. Inhale your medicine. See Figure C.**<br>• Put the mouthpiece between your lips, and close your lips firmly around it. Your lips should fit over the curved shape of the mouthpiece.<br>• Take one long, steady, deep breath in through your mouth. **Do not** breathe in through your nose.
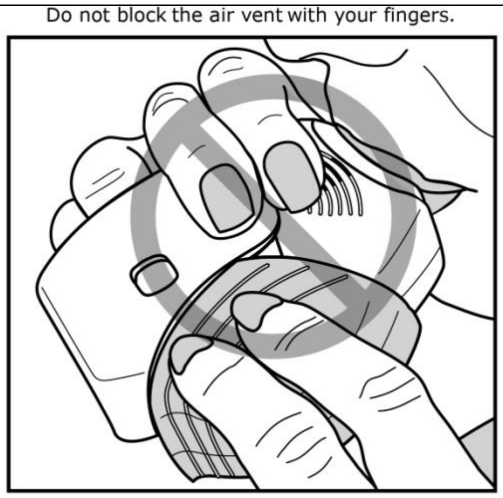<br><br>**Figure D**	• Do not block the air vent with your fingers. **See Figure D**
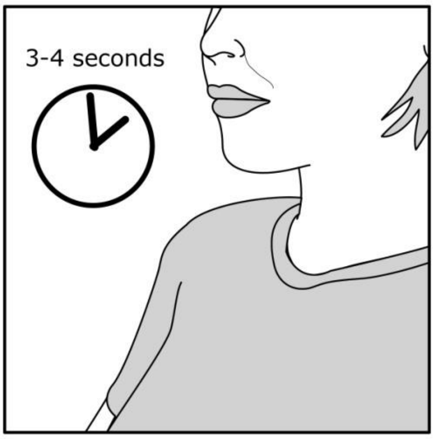<br><br>**Figure E**	• **Remove the inhaler from your mouth and hold your breath for about 3 to 4 seconds** (or as long as comfortable for you). **See Figure E.**

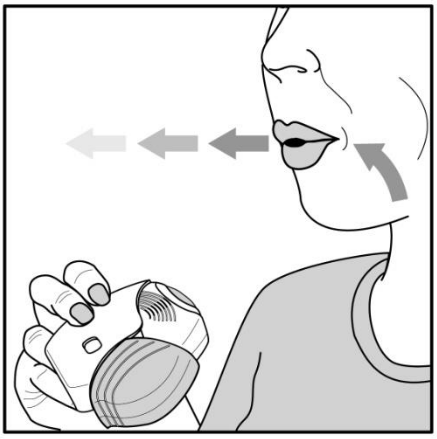<br>**Figure F**	**Step 4. Breathe out slowly and gently. See Figure F.**<br>• You may not taste or feel the dose, even when you are using the inhaler correctly.<br>• **Do not** take another dose from the inhaler even if you do not feel or taste the dose.
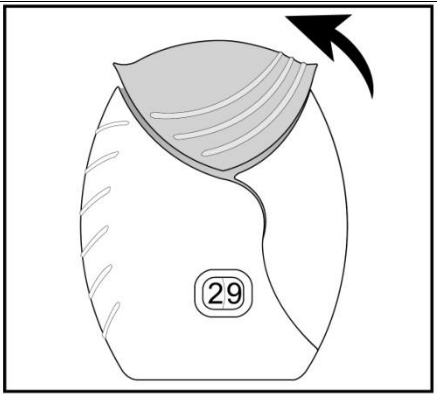<br><br>**Figure G**	**Step 5. Close the inhaler. See Figure G.**<br>• You can clean the mouthpiece if needed, using a dry tissue, before you close the cover. Routine cleaning is not required.<br>• Slide the cover up and over the mouthpiece as far as it will go

Supplementary Table 1. ELLIPTA correct use checklist.

**Steps identified in the ELLIPTA package insert.[3,4]**	**Patient actions to be checked by the trained HCP at: Visit 1 (up to 3 attempts); Visit 2 (1 attempt).**<br><br>**Check YES OR NO to respond to each action/step.**	
**Step 1. Open the cover of the inhaler. See Figure A.[a]**<br><br>Slide the cover down to expose the mouthpiece. You should hear a “click.” The counter will count down by 1 number.	Yes No<br>☐ ☐	Patient slides the cover completely down to expose the mouthpiece until a “click” is heard.
You do not need to shake this kind of inhaler. **Your inhaler is now ready to use.**<br><br>If the counter does not count down as you hear the click, the inhaler will not deliver the dose. Call your healthcare provider or pharmacist if this happens.	Yes No<br>☐ ☐	Patient **does not** shake the inhaler<br><br>(Note: a “Yes” response indicates that the patient **did not** shake the inhaler after the click is heard)
**Step 2. Breathe out. See Figure B.[a]**<br><br>While holding the inhaler away from your mouth, breathe out (exhale) fully.	Yes No<br>☐ ☐	Patient breathes out (exhales) while holding the inhaler away from their mouth.
Do not breathe out into the mouthpiece.	Yes No<br>☐ ☐	Patient **does not** breathe into the mouthpiece.<br><br>(Note: a “Yes” response indicates that the patient **did not** breathe into the mouthpiece)

<table>
<tr><th>Steps identified in the ELLIPTA package insert.[3,4]</th><th colspan="2">Patient actions to be checked by the trained HCP at: Visit 1 (up to 3 attempts); Visit 2 (1 attempt).

Check YES OR NO to respond to each action/step.</th></tr>
<tr><td rowspan="4">Step 3. Inhale your medicine. See Figure C.[a]

Put the mouthpiece between your lips, and close your lips firmly around it. Your lips should fit over the curved shape of the mouthpiece.

Take one long, steady, deep breath in through your mouth. Do not breathe in through your nose.

Do not block the air vent with your fingers. See Figure D.[a]

Remove the inhaler from your mouth and hold your breath for about 3 to 4 seconds (or as long as comfortable for you). See Figure E.[a]</td><td>Yes No
☐ ☐</td><td>Patient places mouthpiece between lips, and closes lips firmly around it.</td></tr>
<tr><td>Yes No
☐ ☐</td><td>Patient takes one long steady deep breath in through their mouth.</td></tr>
<tr><td>Yes No
☐ ☐</td><td>Patient <u>does not</u> block air vent with fingers.

(Note: a “Yes” response indicates that the patient <u>did not</u> block the air vent with their fingers)</td></tr>
<tr><td>Yes No
☐ ☐</td><td>Patient removes inhaler from his/her mouth and holds his/her breath.</td></tr>
<tr><td>Step 4. Breathe out slowly and gently. See Figure F.[a]

You may not taste or feel the dose, even when you are using the inhaler correctly.

Do not take another dose from the inhaler even if you do not feel or taste the dose.</td><td>Yes No
☐ ☐</td><td>Patient breathes out slowly and gently.</td></tr>
<tr><td>Step 5. Close the inhaler. See Figure G.[a]

You can clean the mouthpiece if needed, using a dry tissue, before you close the cover. Routine cleaning is not required.

Slide the cover up and over the mouthpiece as far as it will go.</td><td>Yes No
☐ ☐</td><td>Patient closes the inhaler completely.</td></tr>
<tr><td colspan="3">[a]For figures please refer to the Instructions for Use (Appendix B)</td></tr>
</table>

Appendix C. ELLIPTA inhaler ease-of-use questionnaire.

INSTRUCTIONS: The administrator (investigator or designee) will complete the following questions related to the ELLIPTA inhaler used during this study. Check only **one** response for the question asked.

ELLIPTA inhaler questionnaire (Version A)

Instructions: Please complete the following questions related to the ELLIPTA inhaler that you used during this study. Choose only **one** response for each question.

1. How easy or difficult is it to use the ELLIPTA inhaler?
   □ Very easy
   □ Easy
   □ Difficult
   □ Very difficult

2. How easy or difficult is it to tell how many doses are left in the ELLIPTA inhaler?
   □ Very easy
   □ Easy
   □ Difficult
   □ Very difficult

3. If your **current daily inhaled asthma/COPD medication**[a] was available in the ELLIPTA inhaler, how likely or unlikely would you be to request the medication in the ELLIPTA inhaler from your doctor?
   □ Very likely
   □ Likely
   □ Unlikely
   □ Very unlikely

[a]As relevant; asthma medication for subjects in the asthma study and COPD medication for subjects in the COPD study

ELLIPTA inhaler questionnaire (VERSION B)

Instructions: Please complete the following questions related to the ELLIPTA inhaler that you used during this study. Choose only **one** response for each question.

1. How easy or difficult is it to use the ELLIPTA inhaler?
   □ Very Difficult
   □ Difficult
   □ Easy
   □ Very Easy

2. How easy or difficult is it to tell how many doses are left in the ELLIPTA inhaler?
   □ Very Difficult
   □ Difficult
   □ Easy
   □ Very Easy

3. If your **current daily inhaled asthma/COPD medication**[a] was available in the ELLIPTA inhaler, how likely or unlikely would you be to request the medication in the ELLIPTA inhaler from your doctor?
   □ Very Unlikely
   □ Unlikely
   □ Likely
   □ Very Likely

[a]As relevant; asthma medication for subjects in the asthma study and COPD

medication for subjects in the COPD study

Supplementary references

1. National Asthma Education and Prevention Program. Expert Panel Report 3 (EPR-3): Guidelines for the diagnosis and management of asthma – Summary Report 2007. *J Allergy Clin Immunol* 2007; 120(5 Suppl): S94–138.
2. Celli BR, MacNee W; ATS/ERS Task Force. Standards for the diagnosis and treatment of patients with COPD: a summary of the ATS/ERS position paper. *Eur Respir J* 2004; 23(6): 932–946.
3. GlaxoSmithKline. ARNUITY ELLIPTA (fluticasone furoate inhalation powder), for oral inhalation use. Highlights of prescribing information. Revised May 2018. Available at: https://www.gsksource.com/pharma/content/dam/GlaxoSmithKline/US/en/Prescribing_Information/Arnuity_Ellipta/pdf/ARNUITY-ELLIPTA-PI-PIL.PDF. Accessed: 4 September 2018.
4. GlaxoSmithKline. BREO ELLIPTA (fluticasone furoate and vilanterol inhalation powder), for oral inhalation. Highlights of prescribing information. Revised December 2017. Available at: https://www.gsksource.com/pharma/content/dam/GlaxoSmithKline/US/en/Prescribing_Information/Breo_Ellipta/pdf/BREO-ELLIPTA-PI-MG.PDF. Accessed: 4 September 2018.
